# Too little or too much nocturnal movements in Parkinson’s disease: A practical guide to managing the unseen

**DOI:** 10.1016/j.prdoa.2024.100258

**Published:** 2024-05-25

**Authors:** Jirada Sringean, Ornanong Udomsirithamrong, Roongroj Bhidayasiri

**Affiliations:** a Chulalongkorn Centre of Excellence for Parkinson’s Disease & Related Disorders, Department of Medicine, Faculty of Medicine, Chulalongkorn University and King Chulalongkorn Memorial Hospital, Thai Red Cross Society, Bangkok 10330, Thailand; b The Academy of Science, The Royal Society of Thailand, Bangkok 10330, Thailand

**Keywords:** Parkinson’s disease, Nocturnal movement disorders, Sleep-related movement disorders, Nocturnal hypokinesia, Periodic limb movement disorders, Evening dyskinesia

## Abstract

Nocturnal and sleep-related motor disorders in people with Parkinson’s disease (PD) have a wide spectrum of manifestations and present a complex clinical picture. Problems can arise due to impaired movement ability (hypokinesias), e.g. nocturnal hypokinesia or early-morning akinesia, or to excessive movement (hyperkinesias), e.g. end-of-the-day dyskinesia, parasomnias, periodic limb movement during sleep and restless legs syndrome. These disorders can have a significant negative impact on the sleep, daytime functional ability, and overall quality of life of individuals with PD and their carers. The debilitating motor issues are often accompanied by a combination of non-motor symptoms, including pain and cramping, which add to the overall burden. Importantly, nocturnal motor disorders encompass a broader timeline than just the period of sleep, often starting in the evening, as well as occurring throughout the night and on awakening, and are not just limited to problems of insomnia or sleep fragmentation. Diagnosis can be challenging as, in many cases, the ‘gold standard’ assessment method is video polysomnography, which may not be available in all settings. Various validated questionnaires are available to support evaluation, and alternative approaches, using wearable sensors and digital technology, are now being developed to facilitate early diagnosis and monitoring. This review sets out the parameters of what can be considered normal nocturnal movement and describes the clinical manifestations, usual clinical or objective assessment methods, and evidence for optimal management strategies for the common nocturnal motor disorders that neurologists will encounter in people with PD in their clinical practice.

## Highlights

• Nocturnal and sleep-related motor disorders in PD present a complex picture.
• If untreated, these disorders can impact sleep and overall quality of life.
• Onset can start in the evening, occur during the night, or on waking.
• Problems can arise due to either hypokinetic or hyperkinetic movements.
• This review guides recognition, assessment and treatment approaches.

## 1 Introduction

**Fig. 1:**
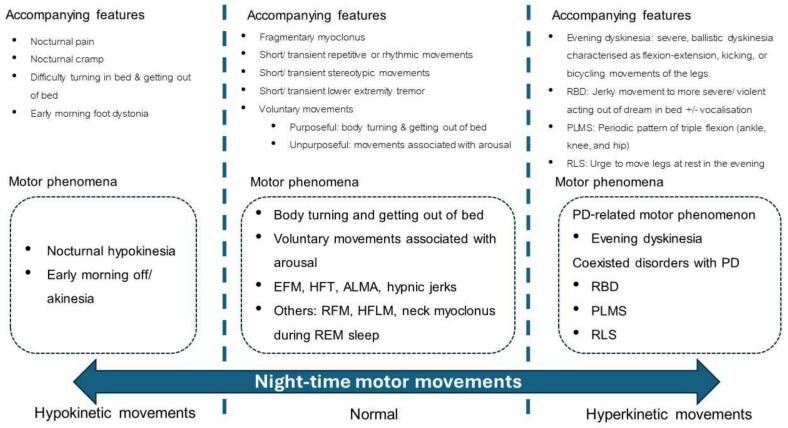
The spectrum of nocturnal motor disorders ranging from those due to hypokinetic movements (left) to those due to hyperkinetic movements (right). ALMA, alternating legs muscle activation; EFM, excessive fragmentary myoclonus; HFT, hypnagogic foot tremor; HFLM high-frequency leg movements; PD, Parkinson’s disease; PLMS, periodic limb movements during sleep; RBD, REM sleep behaviour disorder; REM, rapid eye movement; RFM, rhythmic feet movements; RLS, restless legs syndrome.

Nocturnal and sleep-related motor disorders in Parkinson’s disease (PD) comprise a wide spectrum of manifestations, ranging from moving too little, as in the case of nocturnal hypokinesia/akinesia and early morning akinesia, to moving too much, as seen with different forms of hyperkinesia (Fig. 1). Some of these manifestations are related primarily to PD itself, such as end-of-the-day dyskinesia and early morning dystonia; however, other disorders may coexist with PD, including motor manifestations of rapid eye movement (REM) sleep behaviour disorder (RBD), restless legs syndrome (RLS), or periodic limb movement during sleep (PLMS) [1], [2], [3], [4].

This complex clinical picture of night-time motor symptoms is not easy for practising neurologists to easily understand, particularly when the movements in question generally cannot be observed directly (occurring at night and in the patient’s own home), and so assessment relies on second-hand information relayed by patients or carers which is often selective or limited by recall bias, with the movement problem often only partially witnessed. Recently, ‘visual evidence’ to assist neurologists with the assessment and management of these night-time problems has become an option due to the increasing availability of smartphones that can capture these night-time episodes or the use of video cameras that can be easily installed and set up by an individual with PD or their family members; however, these are not universally available. When assessing nocturnal movement problems, it is important to note that those occurring in people with PD are likely to include ‘normal’ physiological movements, so it is essential that these are defined at the outset in order to set a baseline for what can be considered problematic. Also of note is that, although categorised as ‘nocturnal’ movement disorders, assessment of these problems needs to look at a broader timeline than just focusing on the person’s specific period of overnight sleep, and should start at the end of the day when the last dose of oral PD medication is often taken, right through to morning when they take their first dose of the day or set up an infusion device – a ‘dusk-till-dawn’ approach [2].

If left untreated, nocturnal and sleep-related movement disorders can substantially impact the quality of a person [5], resulting in fragmented sleep, frequent waking and sleep deprivation, which may have knock-on effects in terms of impairment of daytime functional ability and increased daytime somnolence. Similarly, the presence of early morning OFF akinesia is known to affect the overall quality of life for individuals with PD [6]. These observations highlight the importance of correctly identifying, assessing and implementing effective management strategies for nocturnal motor disorders in PD. The complexity of these disorders underscores the need for a comprehensive understanding of their aetiology that can help inform the most suitable treatment approaches. Currently, management remains challenging due to a lack of consensus on optimal therapeutic strategies, but approaches typically involve a combination of pharmacological, non-pharmacological, and adjunctive interventions.

Night-time motor problems in PD are exceedingly complex and are not just limited to problems of insomnia or sleep fragmentation, as is commonly assumed. This review aims to provide practical advice for neurologists when managing these difficult and multifactorial conditions. We start by discussing what can be considered as the parameters of normal physiological movements in sleep and then provide a comprehensive evaluation of nocturnal motor disorders that can occur in people with PD ranging from those experienced at evening time (dusk), during the night when sleeping, and in the morning (dawn). The goal is to enhance physicians’ recognition of their clinical presentation, accurate assessment, and to facilitate selection of appropriate evidence-based management strategies, including both pharmacological and non-pharmacological interventions where appropriate. We hope that this review will lead to better recognition by neurologists of nocturnal motor issues in people with PD which will ultimately result in patients receiving the ‘right’ treatment for their particular disorder.

## 2 Diagnosis of nocturnal motor problems in PD

An accurate clinical history and comprehensive physical examination are fundamental initial steps in the diagnosis of nocturnal motor disorders in people with PD. This should include evaluation of patterns and characteristics of motor behaviour and movement events throughout the evening prior to going to sleep, during the night, and in the morning when they wake up, including the timing of each occurrence, its duration, and its frequency. Additionally, information regarding dream content, eye status (closed or open), sleep onset/offset or sleep–wake pattern disruption, disturbed sleep, daytime sleepiness, and snoring, as well as current medication usage should be documented.

Individuals with PD should be encouraged to use a sleep diary or to collect information about their sleep patterns and the number of awakenings during the night, however the limitations of these tools should be acknowledged as they are typically completed during the day, relying solely on indirect recall of information from patients themselves, their bed partners, or carers, and so may overlook some nocturnal symptoms. Further assessment utilising objective screening tools is recommended to ensure a comprehensive evaluation and accurate diagnosis. Various validated questionnaires are available to evaluate nocturnal motor symptoms and sleep-related disorders. These include the Nocturnal Hypokinesia Questionnaire (NHQ) [7], RBD Screening Questionnaire (RBDSQ) as well as other validated RBD screening or assessment questionnaires [8], [9], [10], RLS Questionnaire [11], Parkinson’s Disease Sleep Scale-2 (PDSS-2) [12], and the Epworth Sleepiness Scale (ESS) [13]. However, the gold standard for objectively investigating sleep and nocturnal movement is video polysomnography (v-PSG), which provides a comprehensive assessment of sleep architecture and physiological parameters [14]. Although a valuable tool, v-PSG is costly and requires trained sleep technicians and specialists for accurate application and interpretation, so it may not be accessible in all settings. In addition, while v-PSG can diagnose most sleep disorders, it may not be suitable for detecting specific nocturnal motor conditions, such as hypokinesia or early-morning akinesia which are among the most common and disabling nocturnal motor disorders in PD.

These limitations highlight the need for alternative, affordable, cost-effective, and practical diagnostic methods that can be easily implemented by neurologists and enable assessment of the full range of nocturnal motor problems. There are now an increasing number of alternative approaches being developed aimed at enhancing accessibility, reducing costs, and facilitating early diagnosis and monitoring, such as the utilisation of wearable devices capable of unobtrusively and continuously monitoring physiological signals during sleep [13]. Examples include actigraphy [15], [16], the NIGHT Recorder [17], wearable PSG [18], or smartwatches [19], which offer the possibility for remote monitoring and assessment. Several wearable devices have been shown to be effective in detecting RBD, including a home-based wearable headband, actigraphy, other types of wearable sensors. In one study, a home-based wearable headband, consisting of 4 soft electrodes for electroencephalography (EEG), electrooculography (EOG), and electromyography at the mentalis muscle, was compared to v-PSG for the detection of RBD. The results indicated that the home-based wearable headband had a sensitivity of 85.7 % and a specificity of 58.3 % in detecting REM sleep without atonia [18]. Using a cut-off of 95 wake bouts per night, actigraphy demonstrated high specificity in identifying RBD (95.5 %) but low sensitivity (26.1 %) [20]. However, sensitivity has been found to increase when actigraphy is combined with other RBD screening methods, such as the RBD questionnaire (95.2 %) [21], or by undertaking deeper analysis using machine learning algorithms, which have demonstrated a high accuracy ranging from 92.9 ± 8.16 % to 100 % [22]. Home video monitoring of sleep behaviour can also be employed, but interpretation of the information captured generally requires input from specialists. A previous study employing automated 3D video analysis of lower limb movements during REM sleep demonstrated that minor leg jerks with a duration of less than two seconds had the highest accuracy (90.4 %) in discriminating isolated RBD from other motor activity during sleep [23]. This finding highlights the potential utility of this approach for home-based RBD detection. These approaches also require full validation and optimization before their integration into regular clinical practice can be considered feasible.

## 3 Normal physiological motor phenomena during sleep and the sleep–wake transition period

In all people, sleep is a dynamic process characterised by distinct stages and physiological changes in muscle tone, brain wave patterns, and eye movements throughout the sleep–wake cycle. There are two phases of sleep: rapid eye movement (REM) and nonrapid eye movement (NREM) sleep, which can be further divided into three stages, N1, N2 and N3 [24]. During normal sleep these phases repeat in a cyclical pattern around 4–6 times each night.

While sleep is often perceived as a state of physical inactivity, a range of physiological motor phenomena naturally occur during the different sleep stages and in the transitional periods between sleeping and waking. These movements range from voluntary actions associated with arousal to more involuntary and stereotypic behaviours. To date, few studies have focused specifically on physiological movements during sleep. A v-PSG study of 100 healthy subjects revealed that voluntary movements associated with arousal are commonly observed during the sleep-wake transition period, such as body turning and getting out of bed [25]. In addition, small, brief involuntary movements or stereotypic actions, with distinct forms can be observed in different sleep stages. These elementary movements can be categorised into three main types: myoclonic, non-myoclonic, and stereotypic movements. Myoclonic movements, characterised by brief, involuntary muscle contractions, are more prominent during REM sleep. In contrast, non-myoclonic movements are more commonly observed during non-REM (NREM) sleep stages. Stereotypic movement patterns, characterised by repetitive and patterned actions, are also observed. The study also showed that physiological movements are most commonly observed in the lower extremities (51.6 %), followed by the upper extremities (36.3 %), trunk (4.8 %), neck (4.7 %), and head (2.6 %), with no significant difference between NREM and REM sleep [25].

According to the International Classification of Sleep Disorders, Third Edition (ICSD-3) [14], certain physiological movements during sleep are classified as isolated symptoms and normal variants. These include excessive fragmentary myoclonus (EFM), hypnagogic foot tremor (HFT), alternating legs muscle activation (ALMA), and hypnic jerks [26], [27]. However, other physiological movements, such as rhythmic feet movements (RFM), high-frequency leg movements (HFLM), and neck myoclonus during REM sleep, are not explicitly classified in the ICSD-3. Understanding the spectrum of physiological movement during sleep is crucial for distinguishing normal sleep patterns from pathological conditions.

### 3.1 The spectrum of nocturnal motor disorders in PD

The clinical presentation of nocturnal motor disorders in people with PD spans a spectrum ranging from reduced movement compared to normal (e.g. nocturnal hypokinesia), normal physiological movements, through to a greater degree of movement than normal (e.g. hyperkinesias) (Fig. 1). Symptoms can also range in severity from mild to severe, and these motor problems are often accompanied by a combination of non-motor symptoms including pain (which can be especially severe on waking), cramping, and paresthesia. Once progressing to the moderate stage, they are already likely to be experiencing motor symptom fluctuations towards the end of the day due to wearing-off of their afternoon or evening dose of oral levodopa. Some may report some slowness of movement when going about their usual early evening activities, in particular tremor or gait difficulties. However, not many of them seek treatment as they may feel that as it is only a few more hours before they go to bed, additional medications may not be required, and maybe hoping that these suboptimal OFF symptoms may subside as they go to sleep. In fact, the opposite is true and these early evening symptoms can make ‘susceptible’ patients more prone to night-time OFF symptoms, especially if they do not take night-time dopaminergic medications, particularly long-acting drugs. In the following sections we will review each of these nocturnal motor disorders focusing on clinical manifestations, the usual clinical or objective assessment methods, and evidence for optimal management strategies.

Moreover, various non-motor symptoms have been found to influence nocturnal motor problems, and impaired sleep quality associated with these nocturnal motor problems also impacts daytime non-motor symptoms. Nocturnal pain, a non-motor symptom, typically occurs concomitantly with night-time OFF symptoms and is associated with sleep dysfunction and dysautonomia [28]. Conversely, PD patients with RBD have been found to exhibit significantly lower cognitive performance across measures of Global Cognitive Functioning, memory deficit, executive functions, attention/working memory, language, and visuospatial abilities. Furthermore, both PD patients with RBD and those with excessive daytime sleepiness demonstrated worse cognitive performance compared to PD patients without sleep disorders during follow-up compared with baseline evaluation [29], [30].

## 4 Moving too little – Nocturnal hypokinesias in PD

The impaired ability of people with PD to move in bed at night (nocturnal hypokinesia) or when they awake in the morning before their usual medication takes effect (early morning akinesia) may be due in part to a continuation of daytime motor PD symptoms, to the wearing-off of dopaminergic medication, and a decline in ability of the dopaminergic system to store dopamine overnight [31].

### 4.1 Nocturnal hypokinesia

**Fig. 2:**
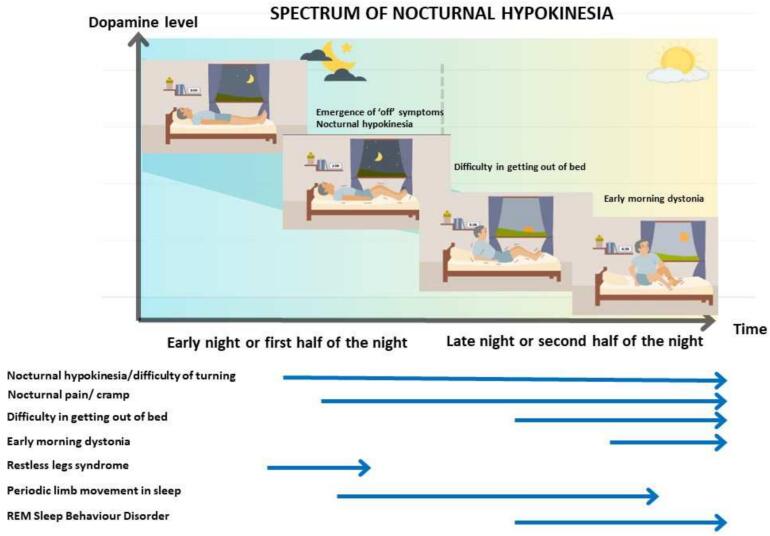
Spectrum of nocturnal hypokinesia and associated manifestations. Individual arrows reflect the period during the night in which the disorder becomes symptomatic.

Nocturnal hypokinesia describes the decreased ability to perform axial rotation and/or trunk flexion in order to turn or change body position in bed [32], [33]. It is considered one of the most troublesome nocturnal symptoms in PD since it can result in impaired sleep quality, which impacts quality of life for both people with PD patients and their carers [34], [35]. Clinical presentation can range from mild symptoms such as early morning dystonia to more severe manifestations including difficulty turning in bed or getting out of bed independently. The majority of individuals with PD tend to adopt a supine position during sleep, and the severity of their symptoms gradually worsens as the night progresses (Fig. 2) [32]. Nocturnal hypokinesia can manifest in *de novo* PD and in the early stages and is associated with bradykinesia, rigidity, axial motor symptoms [32], [36], [37]. Moreover, it is associated with autonomic dysfunction, cognitive impairment, and dosage of dopaminergic medications [36]. Additionally, nocturnal hypokinesia has been identified as a potential predictor for phenoconversion in prodromal group and for development of motor complication in the future in *de novo* PD patients [37], [38]. Nocturnal hypokinesia is recognised as the longest wearing-off period experienced by people with PD and often correlates with experiencing daytime wearing-off [39]. The prevalence of nocturnal hypokinesia in people with PD varies with reports of its occurrence in up to 70 % of subjects. This observed heterogeneity can be attributed to the diverse methodologies utilised for data collection, ranging from questionnaire-based surveys to wearable sensor technologies [40], [41].

There are several validated screening tools for assessing nocturnal hypokinesia including the NHQ [7], selected items from Movement Disorder Society–Unified Parkinson's Disease Rating Scale (MDS-UPDRS) part II (item 2.9 turning in bed, item 2.11 getting out of bed) [42], item 9 from the PDSS-2 (‘Did you feel uncomfortable at night because you were unable to turn around in bed or move due to immobility?’) [12]. The 10-item NHQ has shown good agreement between responses from patients and carers [7]. In addition, wearable sensors (triaxial accelerometer and gyroscope-based devices) such as the NIGHT-Recorder [17], [43] or an actigraphy device can be used to gather objective data. The NIGHT-Recorder is able to assess a range of movement characteristics including speed of rotation, duration, angle, number of rotations, torque of rotation (indicative of reduced ability to turn), and utilisation of multi-step strategies for getting out-of-bed (i.e. initiating movement with the arms or legs) and can discriminate between individuals with advanced PD, those with early-PD and age-matched healthy controls [32], [44], [45]. Studies using the NIGHT-Recorder data have also shown that nocturnal hypokinesia often becomes worse as the night progresses, resulting in significantly fewer rotations, reduced degree of rotation, slower speed, and decreased acceleration during the second half of the night compared to the first half [46].

Evidence supporting both non-pharmacological and pharmacological treatment strategies to manage nocturnal hypokinesia in people with PD are summarised in Table 1. However, due to the lack of head-to-head comparisons between the different medications, determining the optimal first-line treatment remains challenging. Factors influencing the selection of appropriate medications include the occurrence and severity of daytime motor symptoms, availability of treatment options, co-morbidities, possible adverse effects, as well as local reimbursement policies.

**Table 1:** Evidence supporting treatment strategies to manage nocturnal hypokinesia and early-morning OFF/akinesia in people with Parkinson’s disease.

**Treatment strategy**	**Recommended strategy/drug dosage**	**Benefits**	**Possible side effects**	**Efficacy and safety**	**Long-term outcomes**
***Non-pharmacological therapies***					
Strategies for getting out of bed [31], [45], [47]	Multistep strategy, using arms and legs to move the trunk. Mentally rehearsing a movement before attempting it, and breaking a movement down into steps to avoid the need for simultaneous actions with consultant	Improvement in mobility in bed.	−	NA	NA
Bedroom environmental adaptation [48], [49], [50]	Bed rail. Suitable bed height and size. Friction-reducing bedsheets, lightweight quilt, or blanket.	Improvement in mobility in bed.	−	NA	NA
***Pharmacologic therapies***					
*Dopamine agonists*					
Rotigotine transdermal patch [58], [59], [60], [61], [62]	2–16 mg.	Improvement in nocturnal hypokinesia, early-morning OFF/akinesia, pain. Improvement in frequency and degree of turning in bed. Improvement in PDSS-2, modified PDSS, total UPDRS, UPDRS-III, early morning UPDRS-III, UPDRS-axial score, MDS-UPDRS-III and IV, time up and go, NADS, PDQ-8.	Nausea and vomiting Application site reaction Dizziness Other typical dopamine agonist adverse events.	2–4 mg of rotigotine with single drug use, added on to levodopa, and zonisamide improved MDS-UPDRS-III (−11.1 points), IV (−1.1 points), off time (34.6 min), PDSS-2 (−7.3) at 3 months [58]. 10.46 ± 4.63 mg rotigotine improved frequency and degree of turning in bed, total UPDRS, UPDRS-III, UPDRS-axial score, Modified PDSS, NADCS compared to placebo at 12 weeks [59]. 11.83 mg rotigotine improved early morning UPDRS-III (−9.3 points and 46 % of patients improved UPDRS-III by > 30 %), time up and go test (−1.4 sec), mean finger tapping (26.5 taps/min), 61 % of patients improved NADCS by 32 % at 12 weeks [60]. 2–16 mg rotigotine improved early morning UPDRS-III (−3.55 points) and PDSS-2 (−4.26 points), and NADCS (−0.41 points) compared to placebo at 12 weeks. 16 % of rotigotine-treated group decreased dosage to 2 mg due to tolerability concern [62].	weeks.
Ropinirole prolonged release (PRP) [63], [64], [65]	2–24 mg (adjunctive).	Reduction in frequency of nocturnal awakening. Improvement in PDSS, PSQI, ESS, and UPDRS-III.	Risk of impulse control disorders. Risk of sudden onset of sleep. Other typical dopamine agonist adverse events.	Switching of ropinirole intermediated release (PIR) to 17.2 ± 6 mg of PRP improved ON UPDRS-III (−3.1 points), PDSS, PSQI, and ESS at 5–13 weeks. New onset and worsening leg edema and nighttime confusion were reported [63]. Adjunct ropinirole 2–24 mg (19.0 – 19.3 ± 5.73––5.94 mg) improved on time during nighttime and early morning, PDSS compared to placebo at week 24 [64], [65].	5–24 Weeks.
Subcutaneous apomorphine injection and infusion [66], [67]	34.8 ± 6.5 mg (extended nocturnal infusion). 1–10 mg (single injection for rescue therapy).	Reduction of nocturnal awakenings, nocturnal OFF periods, pain, early-morning OFF/ akinesia, dystonia and nocturia. Improvement in frequency, speed, and degree of turning in bed. Improvement in PDSS-2, NADCS, UPDRS- II, III, and IV, and axial score of UPDRS.	An infusion-free gap of at least four hours is required for each 24-hour infusion period. Tolerance. Discomfort. Skin nodule or injection site reaction. Other typical dopamine agonist adverse events.	6 PD with overnight apomorphine improved nocturnal off time, pain, dystonia, and nocturia while 3 placebo improved nocturnal pain, spasm, and sleep disruption [66]. 34.8 ± 6.5 mg, extended nighttime dosage improved in frequency, speed, and degree of turning in bed assessed by wearable sensor and improved PDSS-2, NADCS, UPDRS- II, III, and IV, and axial score of UPDRS [67].	NA
Pramipexole (sustained-release and immediate-release formulations) [68]	1.5 mg/day	Improvement in PDSS-2, NHQ, SCOPA-S, EMO, ESS.	Risk of impulse control disorders. Risk of sudden onset of sleep. Other typical dopamine agonist adverse events.	The mean improvements of PDSS-2 in pramipexole SR and IR were 13.7 points and 14.4 points. At least 50 % reported at least one mild to moderate intensity adverse effect and equal in both formulas [68].	18 weeks
*Monoamine oxidase inhibitor*					
Rasagiline [69], [70], [71]	1 mg	Improvement in sleep quality and excessive daytime sleepiness. May be beneficial for treating early-morning OFF/akinesia by improving axial impairment (rising from a chair). Improvement in ESS. Improvement in sleep maintenance, wake after sleep onset, numbers of arousal, light sleep.	Risk of hallucinations.	Adjunctive double-blind placebo-controlled trial of 1 mg rasagiline and entacapone. Rasagiline improved UPDRS-III (−5.64 points) compared to placebo, while entacapone was not significant compared to placebo (−3.22 points) at week 18 [71].	18 weeks.
*Levodopa*					
Standard or sustained-release levodopa carbidopa [51], [53]	NA	Improvement in nocturnal hypokinesia and possible improvement of early morning akinesia. Increase sleep duration. Improvement of NADCS.	General side effects of levodopa.	Double-blind placebo-controlled cross-over study. Single dose of sustained-release levodopa carbidopa improved NADCS (1.9 ± 0.14) compared to placebo (2.9 ± 0.14) [51]. Double-blind placebo-controlled cross-over study. Morning dose of standard levodopa carbidopa to sustained-release levodopa carbidopa improved morning on time (47 min vs 58 min) [53].	2 weeks
Immediate-release levodopa/carbidopa [52]	200 mg levodopa bedtime	Earlier improvement of nocturnal hypokinesia and early morning off/ akinesia.	General side effects of levodopa and insomnia, nightmare, restlessness, and reflex daytime somnolence.	200 mg levodopa bedtime improved early morning walking time and number of spontaneous moves in beds compared to placebo and divided dose (100 mg bedtime and 100 mg at 3 A.M.) [52].	NA
Sustained-release levodopa/benserazide [54], [55], [72]	450 mg. [300–600 mg] combination with 360 mg. [250–750] standard release levodopa/benserazide.	Improvement in nocturnal hypokinesia, early-morning OFF/akinesia. Stable long-lasting improvement when combined with standard levodopa/carbidopa.	General side effects of levodopa.	Improved of nocturnal akinesia and early morning akinesia severity [54].	24 months
Levodopa/carbidopa intestinal gel infusion [73]	1,412 mg continuous infusion 14.3 h/day.	Improvement of PDSS-2 at 3, 6, 12, 18, and 24 months. Improvement of ESS at 6, 12 months.	Abdominal cramps. Weight loss.	Improvement of PDSS-2, ESS including nocturnal motor symptoms [73].	12––24 months
Dispersible levodopa/carbidopa [56], [74]	NA	May be beneficial for treating early-morning OFF/ akinesia (rescue therapy).	General side effects of levodopa.	NA	NA
Levodopa powder for orally inhalation (CVT-301) [57]	84 mg. orally inhale adjunct to standard first dose levodopa/ carbidopa	May be beneficial for treating early-morning OFF/ akinesia (rescue therapy).	Cough (mild and transient). Nausea. Upper respiratory tract infection. Discoloration of mucus coughed up from the lungs. Should not be taken by patients taking non-selective MAOI, or who have asthma, COPD, other chronic lung diseases.	Randomize, double-blind, placebo-controlled, 2-way crossover study. Better time-to-ON in treatment group (25.0 min) compared to placebo group (35.5 min) with well-tolerated and no notable safety-concerned [57].	NA

COPD, Chronic Obstructive Pulmonary Disease; ESS, Epworth Sleepiness Scale; EOM, early morning OFF; MAOI, Monoamine Oxidase Inhibitor, MDS-UPDRS, Movement Disorder Society–Unified Parkinson’s Disease Rating Scale; N/A, not available; NADS: Nocturnal Akinesia Dystonia Scale; NHQ, Nocturnal Hypokinesia Questionnaire; PDSS-2, Parkinson’s Disease Sleepiness Scale-2; PDQ-8, Parkinson’s Disease Questionnaire 8-item; NA: not applicable, NADCS: Nocturnal Akinesia Dystonia and Cramp Score; PSQI, Pittsburgh Sleep Quality Index; SCOPA-S, Scales of Outcomes in Parkinson’s Disease – Sleep.

Studies undertaken using wearable sensors found that people with PD often use multiple steps to move their body to get out of bed, starting with movement of their arms or legs before truncal rotation [45], therefore rehabilitation training and education around nocturnal movements using protocols for getting out of bed are recommended [31], [45], [47]. Similarly, adapting the bedroom layout to ensure the bed is not too far from the toilet and is of a suitable height and size with a bed railing and a reachable bedside table may be helpful [48], [49]. Studies on the use of friction-reducing textiles for bedsheets and night clothes reported an improvement in bed mobility, reduced dependence on assistance, and better sleep due to less disruption and discomfort compared with use of traditional textiles [50].

Pharmacological treatments for nocturnal hypokinesia are generally based on the concept of continuous dopaminergic delivery (CDD) in order to maintain stable dopamine levels throughout the night and avoid the drop that can occur once the last evening oral medication dose has worn off [31]. Evidence supporting treatment strategies to manage nocturnal hypokinesia in people with PD are summarized in Table 1 [51], [52], [53], [54], [55], [56], [57], [58], [59], [60], [61], [62], [63], [64], [65], [66], [67], [68], [69], [70], [71], [72], [73], [74]. Administration of nocturnal dopaminergic medications needs to be carefully evaluated to avoid potential adverse effects linked to nocturnal dopaminergic stimulation including insomnia, psychosis, and impulse control disorders [75], [76].

### 4.2 Early-morning akinesia

Early-morning akinesia describes difficulty in moving upon waking in the morning, for example getting out of bed, going to the bathroom, and getting on with usual daily activities, and generally arises due to a delay in achieving an ON state following the first dose of PD medication [77], [78]. Early-morning akinesia is a common motor complication in people with PD, with reports of its occurrence in around 60 % of subjects at all stages of the disease [77], [78], and is frequently a precursor to the person experiencing wearing-off of medication effect during the daytime.

Diagnosis and assessment of early-morning akinesia can be undertaken by clinical assessment of the patient and discussion of their morning functional mobility, supplemented with the use of validated questionnaires, such as selected items of the MDS-UPDRS Part IV (motor complications; items 4.4 functional impact of fluctuations and 4.5 complexity of motor fluctuations) [42] and item 14 from PDSS-2 (‘Did you feel tired and sleepy after waking in the morning?’) [12].

Evidence supporting treatment strategies to manage early-morning OFF/akinesia in people with PD are summarized in Table 1, and in many cases overlap with those used for nocturnal hypokinesia. While CR oral levodopa taken at bedtime has been shown to provide relief from the motor symptoms of nocturnal hypokinesia [51], [52], [54], [55], due to its short half-life and limited longer-term bioavailability, it is not generally effective for cases of early-morning akinesia. However, an initial morning dose of standard oral levodopa prior to taking CR levodopa has been shown to alleviate the problem of delayed-onset of clinical response that results in early-morning akinesia [53]. The use of ‘on-demand’ rescue therapies are recommended for early-morning akinesia to ensure subjects are able to rapidly achieve an ON state. These include dispersible levodopa formulations, which takes effect more quickly than tablets or capsules, subcutaneous apomorphine pen injection, or orally inhaled levodopa [56], [57], [79].

## 5 Moving too much – Nocturnal hyperkinesias in PD

### 5.1 Evening or end-of-day dyskinesia

In addition to the emergence of motor OFF symptoms during the night or during sleep, another rare and challenging motor complication that can occur in people with PD is evening or end-of-the-day dyskinesia, a complex form of levodopa-induced diphasic dyskinesia. Distinctive features are severe, ballistic dyskinesia characterized as flexion–extension, kicking, or bicycling movements of the legs which often forces the patient into a reclined position [80], [81]. These phenomena generally occur at the end of each day, typically beginning approximately two hours after the last levodopa dose, are associated with subtherapeutic levels of levodopa [82], and are known to disrupt sleep onset. However, it is critical that clinicians are able to distinguish evening dyskinesia from peak-dose dyskinesia which can commonly occur in the evening due to the cumulative effect of multiple levodopa doses or delayed absorption of levodopa as a result of delayed gastric emptying time. The common clinical features of peak-dose dyskinesia are usually choreic movements and often dystonic posturing. These involuntary movements are common in the cranio-cervical region, and may be initially mild, involving the neck primarily, and less commonly the lips and jaw. They may later spread to involve the trunk and can become increasingly bothersome [80].

The treatment of diphasic dyskinesia with oral dopaminergic medications, catechol-*O*-methyl transferase (COMT) inhibitors, or other medications, such as benzodiazepines, has shown no efficacy. Furthermore, continuation of levodopa into late evening has proven to be ineffective, as it can shift dyskinesia into early morning hours and disrupt sleep. However, there have been reports indicating that DBS of the globus pallidus internus (GPi) and subthalamic nucleus (STN) can reduce evening or end-of-day dyskinesia [83], which is thought to occur either by a direct effect of stimulation or the postoperative reduction of levodopa dosage, or a combination of both mechanisms.

### 5.2 Parasomnias

Parasomnia is defined as a complex behaviour, consisting of an undesirable, physical, or experiential event occurring during sleep. There are three recognised categories: non-REM parasomnias or disorders of arousal, REM parasomnias, and other parasomnias according to the sleep stage when it occurs. REM sleep behaviour disorder (RBD) is the most common aetiology of REM parasomnia and typical behaviours during RBD episodes include abnormal vocalisations (often loud and uncharacteristic of the person) and jerky movements of the body which usually involve distal extremities [84], [85], [86]. A series of repetitive limb jerks is often followed immediately by more dramatic, and often violent, motor activity, such as punching, flailing around or jumping out of bed. These limb movements often occur in conjunction with impaired bed turning, the primary manifestation of nocturnal hypokinesia [31], [32]. Also of note is that people with idiopathic/isolated RBD, a known risk factor for the subsequent development of PD, have been found to exhibit impaired turning ability in terms of increased duration of turning and decreased mean and peak angular velocity compared to controls when assessed using mobile technology [87].

People with RBD will commonly have altered dream mentation, typically involving violent themes, such as being attacked or chased by people or animals. This can translate into complex motor actions including vivid dream enactment behaviours such as grabbing, biting, or kicking (rarely observed as more than a simple jerky movement), that can be frightening to experience and may lead to injury for the person with PD or their bed partners [85], [88]. During these episodes, the person’s eyes are typically closed and following the episode, they are usually coherent, oriented, and able to recall the dream content [14], [89].

The ICSD-3 consists of repeated episodes of sleep-related vocalisation and/or complex motor behaviours that have been documented by v-PSG as occurring during REM sleep or based on a clinical history of dreaming, and are unable to be explained by other sleep disorders [14], [90]. The v-PSG can be used to assess electromyography (EMG; a measurement of muscle tension) and electroencephalography (EEG) abnormalities during REM sleep and is the gold standard for RBD diagnosis. However, the information that can be derived from v-PSG is not limited only to the assessment of sleep stage or EEG/EMG data, it can also provide evidence of motor phenomenologies through video monitoring. In addition, EMG findings from limbs may provide crucial information that may help with diagnosis, understanding the evolution of RBD, and developing future treatment. This is an area in which artificial intelligence may have a potential role in terms of automating and interpreting complex V-PSG data [91].

When v-PSG is unavailable, or for large-scale epidemiological studies, a range of validated screening tools are available that have high specificity, such as the RBD Screening Questionnaire (RBDSQ) [9], RBD Hong Kong Questionnaire (RBD-HK) [8], RBD Single Question Screen (RBD1Q) [10], Mayo Sleep Questionnaire (MSQ) [92], and the Innsbruck RBD Inventory Questionnaire [93]. However, these have certain limitations in clinical practice, for example, some patients may not be able to complete the questionnaire accurately due to lack of awareness of parasomnia symptoms, false positive responses from other conditions that mimic RBD, or the lack of presenting symptoms, which is common. Evidence supporting non-pharmacologic and pharmacologic treatment strategies to manage REM sleep behaviour disorder in people with PD are summarised in Table 2 [94], [95], [96], [97], [98], [99], [100], [101], [102], [103], [104], [105].

**Table 2:** Supporting evidence for treatment strategies to manage Rapid Eye Movement sleep behaviour disorder in individuals with Parkinson's disease.

**Treatment strategies**	**Recommended strategy/drug dosage**	**Benefits**	**Possible side effects**	**Efficacy and safety**	**Long-term outcomes**
***Non-pharmacological therapies***					
Bedroom safety environment	Removing potentially dangerous objects. Installing bedrails. Placing pillows or other soft items between patient and hard structures Sleeping in separate bed from partner.	Patient and bed partner safety.	−	NA	NA
Promote sleep hygiene	Regular sleep schedule/ pattern Avoid caffeine & alcohol after lunch time. Avoid bright light before bedtime 1–2 hrs. Avoid large meal or exercise before bedtime 2–4 hrs. Quiet, ventilated, and dark bedroom environment Increase daytime outdoor activity. Limit daytime napping. Avoid prolonged bed rest during non-sleeping hours.	Improvement in sleep quality.	−	NA	NA
OSA treatment	CPAP. Sleeping in a lateral position. Use of a mouth guard.	Improvement in sleep quality. Reduction in nocturnal motor behaviors.	Discomfort	NA	NA
Reduce potential aggravating factors [94], [95]	Reduction or discontinuation of SSRIs, SNRIs, tricyclic antidepressants, acetylcholinesterase inhibitors, caffeine, or alcohol.	Reduction in their impact on RBD.	Recurrent or worsening of comorbidities or disorders.	NA	NA
***Pharmacological therapies***					
Clonazepam [96], [97], [98]	0.5–2 mg.	Increase in total sleep time, sleep efficiency, N2, N3, and decreased waking after sleep onset. May be beneficial in reducing phasic EMG activity during REM sleep.	Sedative effective extend to morning or daytime. Falls. Risk of addiction. Worsening OSA. Rebound RBD in abrupt withdrawal.	Clonazepam partially improved subjective evaluation of RBD severity but not objective PSG measurement of RBD severity and atonia index. Residual symptoms were common among RBD patients despite treatment. Improvement of sleep quality, NREM sleep structures, frequency of violent/ aggressive dreams and sleep related injury. Minor adverse events associated with daytime somnolence	Effect on RBD behaviors, sleep quality and sleep structures remained approximately 2.5 years of therapy.
Melatonin [99]	3–12 mg (monotherapy or adjuvant with clonazepam).	Reduction in RBD-related injuries. Improvement in subjective night-time sleep.	Headache. Morning sleepiness. Light-headedness. Fatigue.	No differences in RBD events between treatment and placebo groups during the 8 weeks of treatment intervention. However, sleep-onset latency was improved in treatment group. No report of major AEs	NA
*Other alternative potential RBD treatments*					
Ramelteon [100]	8 mg.	Reduction in RBD events. Improvement in subjective night-time sleep. Improvement in PD motor symptoms.	Daytime somnolence. Nausea. Light-headedness. Delirium. Worsening constipation.	Ramelteon reduced probable REM sleep behaviour disorder symptoms and improved sleep quality in PD patients during 12 weeks of treatment. No report of major AEs	NA
Memantine [101]	20 mg.	Reduction in the nocturnal movements and/or vocalizations in PDD and DLB.	Bradycardia. Nausea.	Memantine reduced probable REM sleep behaviour disorder in patients with DLB and PDD during the 24 weeks of treatment compared to placebo. Memantine is well tolerated in DLB and PDD patients.	NA
Rivastigmine patch [102]	4.6 mg.	Reduction in the mean frequency of RBD episodes recorded by bed partners.	Minor peripheral cholinergic action.	Rivastigmine is more effective than placebo in reducing RBD frequency in PD patients with treatment refractory RBD during the 3-week treatment. Minor AEs include orthostatic hypotension	NA
Rotigotine patch [103]	12.36 ± 4.27 mg/day.	Improvement in subjective severity of RBD symptoms (assessed by RBD-HK) and RBDSS score (based on V-PSG). Increase in total sleep time, decreased PLMS index.		Rotigotine improved RBD symptoms, sleep quality and motor symptoms in PD patients during the 12 weeks treatment. No report of major adverse events	NA
Safinamide [104]	50 mg.	Improvement in subjective severity RBD symptoms assessed by RBD-HK (dream-related movement and falling out of bed). Reduction in PDSS-2, UPDRS part II, III. Increase in total sleep time. Improvement in tonic and phasic submental EMG activities and REM density.	Increased risk of dyskinesia.	Safinamide improved subjective and objective outcomes of RBD and sleep quality in PD patients during the 3 months treatment. No report of major adverse events.	NA
Sodium oxybate [105]	6.55 g., in treatment resistant RBD	Reduction in monthly RBD episodes.	Anorexia. Anxiety. Increased sweating. Brain fog.	Sodium oxybate showed more reduction in number of monthly RBD episodes according to a diary and RBD activity per V-PSG compared to placebo during the 4 weeks treatment. The adverse events were more common in treatment group which resolved with dose adjustment.	NA

CPAP, Continuous Positive Airway Pressure; EMG, Electromyography; N2 & N3, Non-Rapid Eye Movement sleep stage 2,3; NA, not applicable; OSA, Obstructive Sleep Apnea; PDSS-2, Parkinson’s Disease Sleepiness Scale-2; PLMS, Periodic Limb Movement During Sleep; RBD, Rapid Eye Movement (REM) Sleep Behavior Disorder; RBD-HK, Rapid Eye Movement (REM) Sleep Behavior Disorder Questionnaire-Hong Kong; RBDSS, RBD Severity Scale; SSRIs. Selective Serotonin Reuptake Inhibitors; SNRIs, Serotonin Norepinephrine Reuptake Inhibitors; UPDRS: Unified Parkinson’s Disease Rating Scale; V-PSG, video polysomnography.

## 6 Periodic limb movement during sleep and restless leg syndrome

Although some epidemiological and subjective evidence suggests there may be an overlap between the conditions known as periodic limb movements during sleep (PLMS) and restless leg syndrome (RLS; also known as Willis–Ekbom disease), other studies using v-PSG data suggest that they are distinct phenomena [106]. PLMS is reported to occur 4–11 % of the general population and in [107], with much higher prevalence reported in people with PD (almost 60 %) which is thought to increase with disease severity [108]. Studies have reported a wide-ranging prevalence of RLS in people with PD of up to 50 % which may vary depending on whether they are drug-naïve or receiving dopaminergic medication [109], [110], [111].

Periodic limb movements during sleep (PLMS) are characterised by a cluster of four or more repetitive, periodic, and highly stereotypical limb movements, primarily involving lower extremities [14]. This movement typically includes a rhythmic extension of the big toe and dorsiflexion of the ankle and occasional flexion of the knee and hip which may be associated with arousal [14], [112]. An index (number of PLMS per hour of sleep) greater than 5 for the entire night denotes a pathological condition. The prevalence of PLMS in drug naïve PD patients has been found to be similar to that of the general population but is higher among treated PD patients. PLMS demonstrated a positive correlation with PD severity as defined by the UPDRS part II, III, and with camptocormia [108], [113]. PLMS is also associated with subjective sleep complaints despite the absence of objective sleep structure parameters [108].

RLS is defined by the ICSD-3 and International Restless Legs Syndrome Study Group (IRLSS) [14], [90], [114] and characterised by the following set of related characteristics: (1) unpleasant sensations in the legs; (2) during rest or inactivity or in bed; (3) partially or totally relieved by movement; (4) in the evening or night rather during the day; and (5) that is not due to another medical or behavioural condition. There may also be accompanying movements as a result of patients trying to relieve their discomfort which is important information that may aid clinical diagnosis. The reported prevalence of RLS is around 50 % in PD population studies [115], [116], [117], [118]. The diagnosis of RLS in PD, as in the general population, is based primarily on clinical interview and evaluation but this can be supplemented with the sensory component of the suggested immobilisation test (SIT) test showing an increase in sensory leg discomfort [109], [119].

In terms of treatment, there are currently no clinical practice guidelines specifically for the treatment of PLMS in people with PD, therefore, it is recommended that the same non-pharmacological and pharmacological therapies as used for RLS are considered in these cases (Table 3) [120], [121], [122], [123], [124], [125], [126], [127], [128], [129], [130], [131], [132], [133], [134], [135], [136], [137], [138], [139], [140], [141], [142], [143], [144]. In the case of RLS, evaluation of treatment strategies in people with PD currently lacks well-designed studies that can direct treatment selection, so general RLS treatment guidelines are usually adopted.

**Table 3:** Strategies for the management of restless legs syndrome (RLS) and periodic limb movements during sleep in individuals diagnosed with Parkinson's disease: a review of treatment options.

**Treatment strategies**	**Recommended strategy/drug dosage**	**Benefits**	**Possible side effects**	**Efficacy and safety**	**Long-term outcomes**
***Non-pharmacological therapies***					
*Lifestyle interventions*					
Promote regular exercise [120], [121]	Dependent on each patient’s condition (disease severity, economic status, personal acceptance, convenience) for example yoga, walking, or lower body resistance exercise.	Improvement in IRLS scores. Improvement in sleep quality.	Fatigue.	Active exercise reduced RLS severity score by 39 % compared to lifestyle interventions (cigarette and alcohol cessation, avoidance of excessive caffeine, and proper sleep hygiene) which reduced severity score by 8 % during the 12 weeks study [120]. Yoga group demonstrated significantly greater improvement than controls (Educational Film) in several domains of sleep quality, greater reductions in prevalence of insomnia, and greater increases in average sleep duration during the 8 weeks study [121]. Minor AEs including knee pain and muscle soreness in active exercise group.	NA
Pneumatic compression [122]	Wear the therapeutic device at 40 cmH_2_O inflated for 5 s per minute for 1 h prior to the onset of symptoms.	Improvement in IRLS scores. Improvement in quality of life, sleepiness (ESS), and fatigue.	Discomfort.	Therapeutic PCD resulted in greater improvements in the means of all measured variables (IRLS scores, ESS) over the use of sham devices during the 4 weeks treatment. None experienced adverse reactions related to PCD use.	NA
Repetitive transcranial magnetic stimulation (rTMS) [123], [124]	5 Hz applied to the supplementary motor area: one session every 3 days for a total of 10 sessions [123]. 15-Hz rTMS stimulation of the leg motor representative area of the frontal cortex for 14 days [124]	Improvement of IRLS scores [123].Improvement of IRLS scores and improvement in sleep quality (PSQI), anxiety (HAM-A) and depression (HAM-D24) [124].	Transient headache. Local discomfort at stimulation area. Dizziness. Ipsilateral lacrimation. Generalized seizure (rare).	Improvement of IRLS scores over sham stimulation after 5th and 10th sessions [123]. rTMS treatment, improve RLS symptoms, anxiety and depression over sham stimulation [124].	Ongoing effects up to 2 weeks in most of the patients [123]. Curative effect sustained for 2 months [124]
Standard acupuncture [125]	12 acupoints.	Improvement in sleep quality (movement activity during sleep, during first hour, and ESS).	Pain. Discomfort.	Standard but not randomly selected acupuncture points reduced abnormal nocturnal leg activity and early sleep activity significantly in week 2, week 4, and week 6 as well as improvement in the IRLS scores and ESS.	NA
Vibration pads [126]	Pads applied to calf muscles for 35 min each night and during RLS attacks	Improvement of sleep quality.	Discomfort	Vibrating pad was more effective than sham pad in improving The Medical Outcomes Study Sleep Problems Index II scores during the 4 weeks treatment. However, vibration therapy failed to significantly improve scores on the RLS-QoL or IRLS scales.	NA
Movement therapy [127]	Counter strain manipulation: moving the body or limb into a position that reduces tenderness in the point and maintaining that position for ninety seconds.	Improvement of IRLS scores.	Discomfort	Counter strain manipulation for 4 weeks significantly improved IRLS scores over sham therapy, accounting for improvement of 42·2% in the active group compared to 8·7% in the controls.	NA
*Surgery*					
Deep brain stimulation [128], [129], [130]	STN target.	Improvement in IRLS scores. Reduction in dopaminergic medications.	General side effects of DBS. Emergence of RLS (rarely).	Improvement in RLS severity (IRLS scores) [128], [129], sleep quality and quality of life (RLS QoL) [128]. 30–43 % had complete or nearly complete resolution of the symptoms [128], [129]. Activated contacts located in the central sensorimotor region of the STN relieved RLS symptoms while activated contacts in the inferior sensorimotor part of the STN or in the substantia nigra induced RLS symptoms [128]. Improvement of NMSS particularly sleep domain [129].	12 months [128], [129]
***Pharmacological therapies***[131]					
*Iron supplementation*					
Ferrous sulfate [132]	Ferrous sulfate 325 mg and vitamin C 100 mg, twice per day.	Maybe beneficial in improvement of RLS symptoms.	Worsening constipation. Nausea.	Oral ferrous sulfate 325 mg (65 mg elemental iron) and vitamin C 100 mg twice daily for 12 weeks were more effective than placebo for treating RLS (improvement of IRLS score) for patients with serum ferritin 75 µg/l. Ferric carboxymaltose 1000 mg was more effective than placebo for the treatment of moderate to severe RLS in patients with serum ferritin levels < 300 µg/l and transferrin saturation < 45 %. Efficacy was reported at 4 and 6 weeks after treatment.	Improvement of RLS symptoms up to 30 weeks post treatment for IV iron.
*Dopamine agonists*					
Pramipexole [133], [134]	0.25–0.75 mg/day.	Improvement in night-time RLS symptoms. Improvement in overnight motor and non– motor symptoms. Reduction in pain.	Risk of augmentation. Risk of sleep attack. Other general dopamine agonist adverse effects.	Pramipexole was more effective than placebo to improve RLS symptoms, sleep quality and QoL during 12––26 weeks of treatment. Pramipexole was well-tolerated with similar withdrawal rate due to AEs compared to placebo.	NA
Ropinirole [135], [136]	0.78–4.6 mg/day.	Improvement in night-time RLS symptoms. Improvement in overnight motor and non– motor symptoms.	Risk of augmentation. Other general dopamine agonist adverse effects.	Ropinirole was more effective than placebo to improve RLS symptoms, sleep quality and QoL during 12––26 weeks of treatment. The AEs reported with ropinirole were consistent with those previously reported with other dopaminergic agonists.	NA
Rotigotine [137], [138]	2–3 mg/day.	Improvement in night-time RLS symptoms. Improvement in overnight and early-morning motor performance. Reduction in nocturia.	Risk of augmentation Application site skin reaction. Headache. Other general dopamine agonist adverse effects.	Rotigotine transdermal patches were more effective than placebo to improve RLS symptoms, sleep quality and QoL. The efficacy was maintained throughout the 6 months period of treatment. The AEs were dose-dependent manner however were lower than reported for other non-ergot oral dopamine agonists despite the longer duration of the rotigotine trial.	NA
*Alpha 2 delta ligands*					
Gabapentin [139], [140]	800 mg/day in divided doses (200 mg/day in patients undergoing hemodialysis) [139]. 600–2,400 mg/day in divided doses [140]	Improvement in RLS symptoms and reduction in pain [139]. Improvement in IRLS scores, PLMS index, sleep quality (PSQI) and sleep structures (sleep time, sleep efficiency, and slow wave sleep), and reduction in pain [140].	Dizziness. Sleepiness. Peripheral edema.	Gabapentin effectively improved RLS symptoms, sleep outcomes and RLS related pain compared to placebo. The AEs were more common in treatment group however, no significant differences were found in the particular rate of any of these adverse effects [140].	NA
Gabapentin enacarbil [141], [142]	1,200 mg, once daily.	Improvement in RLS symptoms. Reduction in pain	Dizziness. Sleepiness.	Gabapentin enacarbil significantly improved RLS symptoms, sleep quality, RLS related pain and QoL compared to placebo and was generally well tolerated during 2–12 weeks of treatment [142].	NA
Pregabalin [139]	150–450 mg/day, 1–3 h before bedtime.	Improvement in night-time RLS symptoms. Improvement in subjective night-time sleep. Reduction in pain.	Dizziness. Sleepiness. Two case reports of pregabalin-induced dyskinesia.	Pregabalin was more effective than placebo for improving RLS symptoms during 6 weeks of treatment, the response was dose-dependent with doses of ≥ 150 mg/day would provide the greatest response to drug. AEs were more common in treatment group, most were mild.	NA
*Opioids*					
Oxycodone or prolonged released oxycodone naloxone [143]	15.9 mg/day (oxycodone) or oxycodone 5–40 mg plus naloxone 2.5–20 mg, twice per day. (efficacious in severe treatment resistant RLS)	Improvement of leg discomfort, urge to move. Improvement of IRLS scores Improvement in PLMS index, number of awakenings, and sleep efficiency.	Risk of addiction. Constipation. Fatigue. Sleep-related respiratory problems. Pruritus. Hallucinations. Urinary retention.	Prolonged release oxycodone–naloxone was effective for improving RLS symptoms, sleep quality and QoL compared to placebo in patients with severe RLS insufficiently treated with first-line drugs. The efficacy sustained through the 40-week extension phase. The AEs were more common in treatment group; however, the tolerability was proportional to those reported in first-line dopaminergic treatments.	NA
*Benzodiazepines*					
Clonazepam [144]	0.5–1.5 mg/day not recommended as first line due to limited well established evidence.	Unknown benefit but PD patients who use clonazepam have less PLMS and less daytime sleepiness.	Sleepiness. Risk of fall. Risk of addiction. Worsening OSA.	Subjective improvements of restlessness and dysesthesias Reduction of periodic leg movements in sleep (PMS) index	NA

AEs, Adverse events; ESS, Epworth Sleepiness Scale; HAM-A, Hamilton Anxiety Rating Scale; HAM-D24, Hamilton Depression Rating Scale-24; IRLS, International Restless legs Scale; NA, not available; NMSS, Non-Motor Symptoms Scale for Parkinson's Disease; OSA, Obstructive Sleep Apnea; PCD, Pneumatic compression; PLMS, Periodic Limb Movement during Sleep; PSQI, Pittsburgh Sleep Quality Index; QoL, Quality of Life; STN, Subthalamic Nucleus.

## 7 Conclusion

Addressing nocturnal motor disorders in people with PD is crucial for enhancing overall quality of life for both them and their caregivers. In terms of diagnosis, challenges persist, including limited access to the most effective diagnostic tools, such as v-PSG, highlighting the need for more affordable, practical diagnostic methods. It is hoped that the continued incorporation of wearable devices or sensors to support objective assessment of nocturnal movements will help address this and improve diagnostic accuracy. Current treatment strategies for nocturnal motor problems vary depending on the specific underlying issues and may include dopaminergic medications, other non-dopaminergic pharmacological interventions, as well as non-pharmacological management approaches. Further studies to understand the underlying mechanisms of nocturnal motor disorders and to explore novel, more effective, treatment strategies should continue to be undertaken so that we can improve the lives of people with PD.

## Credit authorship contribution statement

**Jirada Sringean:** Writing – original draft, Conceptualization. **Ornanong Udomsirithamrong:** Writing – original draft. **Roongroj Bhidayasiri:** Writing – review & editing, Validation, Project administration, Conceptualization.

## Declaration of competing interest

The authors declare that they have no known competing financial interests or personal relationships that could have appeared to influence the work reported in this paper.
